# Exploratory values for venous blood gas analysis of clinically healthy Hermann’s tortoises (*Testudo hermanni*)

**DOI:** 10.3389/fvets.2026.1816904

**Published:** 2026-06-02

**Authors:** Niccolò Pagnini, Dario Ciappi, Anna Pasquini, Marco Bonazza Foselli, Ilaria Lippi, Francesca Perondi, Alessandro Vetere

**Affiliations:** 1 Ambulatorio Veterinario Galciana, Prato, Italy; 2 Azienda Ospedaliero-Universitaria (AOU) di Parma, Parma, Italy; 3 Department of Veterinary Medical Sciences, University of Pisa, Pisa, Italy; 4 Ambulatorio Veterinario Dalmazia, Florence, Italy; 5 Department of Veterinary Science, University of Parma, Parma, Italy

**Keywords:** blood gas analysis, exploratory values, reptile medicine, *Testudo hermanni*, venous blood

## Abstract

**Background:**

Hermann’s tortoise (*Testudo hermanni*) is included in the red list of endangered species by the International Union of Conservation of Nature (IUCN) and is classified as near-threatened (NT); medical information and knowledge of this species is critically important for preserving the population. Blood gas analysis is being increasingly studied in veterinary medicine, but its main application in chelonian medicine is to assess the health status of marine chelonians; it is not often used for tortoises in field conservational studies or client-owned pet care.

**Objective:**

The aim of this study was to obtain exploratory values for pH, pCO₂, pO₂, ctHb, sO₂, Hct, cK^+^, cNa^+^, cCa^++^, cCl^−^, cGlu, cLac, cBase, mOsm, cHCO₃^−^, ctCO₂ and anion gap for venous blood from clinically healthy *Testudo hermanni* using a Radiometer™ ABL735GLAXP® blood gas analyzer to serve as a starting point for further studies.

**Methods:**

Samples were collected from 38 healthy individuals at both the end of July and the end of September; blood gas tests were performed immediately after sample collection. The data were analyzed to verify the distribution and obtain the exploratory values.

**Results:**

The values obtained were similar to those reported in the literature for analytes such as pH, cK^+^, cNa^+^, cCl^−^, and mOsm; the cGlu value differed from those reported in the literature. For the remaining analytes, reference intervals for *Testudo hermanni* were unknown and were compared to values obtained for other chelonian species.

**Conclusion:**

Exploratory values for venous blood gas analysis were obtained; those values can be used to further investigate this type of diagnostic test and expand the knowledge and methods at the disposal of tortoise clinicians.

## Introduction

Blood gas analysis, both arterial and venous, has become standard in both veterinary and human hospitals (1), especially in critical and emergency care. It is a rapid test that requires a small amount of blood (2), can be performed in the field with portable equipment, and does not require specific laboratory procedure training to be used effectively (3), making it a good choice for both wildlife health monitoring and rapid health assessment in primary care.

Blood gas analysis in reptile medicine is primarily used for rapid assessment when captured reptiles are checked and immediately released. Studies on both arterial and venous samples have been conducted, although venous samples are the most prominent in the literature, probably because of difficulties in conducting arterial sampling and the lack of a standardized method. Venous blood gas analysis has been performed for various taxa, ranging from squamates (4, 5) to both marine and terrestrial chelonians (6–12). Research studies use this analysis both in conjunction with other hematological analyses for comparison purposes (13) and as a stand-alone test to check acid–base balance and analytes such as lactate, which is regarded as a stress indicator (14). It has often been used in health assessments of marine chelonians to investigate parameters that could be used as prognostic indicators in turtles that are stranded, caught in fish nets, or stunned by cold temperatures (8, 9, 11, 12). Blood gas analysis remains an underdeveloped tool but could prove to be useful to obtain specific hematological values such as acid–base balance and lactate and ionized calcium levels, which are reported and investigated as prognostic factors (15, 16), or to aid in the identification of pathological conditions where total calcium assessment could be inaccurate or not sufficient on its own (17–19).

Hermann’s tortoise is endemic to the southern Mediterranean area (20, 21), where it is also kept as a pet. The International Union of Conservation of Nature (IUCN) classified *Testudo hermanni* as Endangered (EN) (47), primarily due to declining wild populations in Mediterranean areas (22). Advances in tortoise medicine are critically important for preventing a decrease in the wild population and enhancing the diagnostic possibilities of clinicians who work with this type of patient. Compared with mammals, tortoises show very few signs during clinical examination, increasing the relevance of clinical pathological parameters because they are likely to be more useful for determining early pathological status. Other studies have investigated the clinical pathology of this reptile (23–26), but few have tested blood gas parameters (13). With respect to marine chelonians, terrestrial chelonians could benefit from blood gas analysis as a rapid tool to estimate stress levels, determine early pathologic statuses, and aid in the definition of prognosis, but the literature lacks species-specific data and evaluations.

Therefore, the aim of this study was to investigate venous blood gas parameters of clinically healthy *Testudo hermanni* during the summer and obtain an exploratory reference value for those parameters to be used as a foundation for future studies.

## Materials and methods

### Ethics statement

All the procedures of this study were approved by the Ethics and Scientific Committee of Pisa University (Protocol N. 5,843). The owners signed an informed consent form for the inclusion of their animals in the study.

### Animals

Forty-two tortoises (*Testudo hermanni*) were included initially in this study, of which thirty-eight were sampled.

To be included, a subject was required to weigh more than 450 g, have a curved carapace with a length of ≥15 cm, and be at least 8 years old. Every animal included was born and raised in captivity, and they were kept in three separate groups, two of which were in Borgo San Lorenzo (province of Florence, Tuscany, Italy) and one of which was in Poggio a Caiano (province of Prato, Tuscany, Italy). All tortoises were kept in outside enclosures, exposing them to natural sunlight and environmental temperature, and all of them hibernated regularly and were fed with autochthonous plants growing inside their enclosures, supplemented with green and red leafy vegetables (including chicory, endive, escarole, and romaine lettuce). The tortoises included in this study were client-owned, ensuring that every patient’s age was correctly determined.

Before the samples were taken, all tortoises underwent clinical examination, and only those judged healthy underwent the blood sampling procedure. Every patient was handled with a disposable glove, which was discarded after every tortoise was examined. The carapace, plastron, every patch of visible skin, and the eyes, nostrils, tympanic area, limbs, and cloacal region were inspected. The oral cavity was then opened and inspected, and the prefemoral region was palpated to investigate the presence of abnormalities in the coelomic cavity. The temperature of the animal was measured with an infrared thermometer aimed at the prefemoral fossa area (12, 39).

Only the clinical examination was included in the evaluation of the patient, as described in the inclusion criteria, as fecal exams were routinely performed by the owner and were not repeated, and other blood tests were not performed.

Four tortoises were deemed unhealthy after the clinical evaluation and were excluded from this study.

### Subjects

Thirty-eight tortoises were included in this study and were selected using the previously explained criteria; six subjects were males, and the other 32 were females. The age of the tortoises varied from 8 to 50 years, with a mean value of 20 years; the curved carapace length varied from 16 cm to 27 cm, with a mean value of 21; and the weight varied from 450 g to 2,375 g, with a mean value of 970 g.

### Environmental conditions

After transfer to the study facility, the tortoises were housed outdoors in single enclosures with access to fresh water, adequate shelter, and direct sunlight exposure. Physical examinations and blood sampling were conducted before and after housing. The temperature range for the sampling day at the end of July was a maximum of 31 °C, a minimum of 20 °C, and a mean of 25 °C; the temperature range for the sampling day at the end of September was a maximum of 30 °C, a minimum of 18 °C, and a mean of 24 °C. The group of tortoises sampled in July consisted of 17 animals, whereas the group sampled in September included 21 tortoises.

All the procedures were performed in the first half of the afternoon.

### Sample collection and blood gas analysis

Tortoises that were eligible for the study underwent the following blood sampling procedure: every tortoise was manually restrained without sedation by two operators; the first operator contained both of the tortoise’s forelegs outside of the carapace by blocking the elbows on the bridge; and the second gently pushed the tortoise’s head in the retracted position and performed blood sampling.

The sampling site was disinfected with a povidone-iodine swab, and blood samples, varying between 0.2 mL and 1 mL, were collected from the dorsal cervical sinus using disposable sterile plastic syringes with a 23-gauge needle (Insu/Light, Rays). The syringes were previously heparinized by three cycles of aspiration and subsequent ejection of 1 mL of heparin solution (Eparina Vister, Teva). A final fourth cycle of air aspiration and ejection was performed to further remove the excess heparin inside the syringe.

If lymph contamination was suspected during sampling, the syringe was immediately discarded, and a second attempt at blood sampling was started. None of the samples exceeded 1 mL of blood.

After sampling, the first operator applied gentle pressure on the sampling site to minimize the possibility of hematoma, while the second operator proceeded to the sampling process. The samples obtained were immediately transported to the laboratory, which was in another room of the same facility, in which blood gas analysis was performed after approximately 5 min. Immediately after sampling, the blood was checked for air bubbles and, if present, they were immediately expelled. The tip of the needle was inserted into a rubber cap to prevent air from entering the syringe. No other precautions were taken during sample transport to the laboratory, as the environment and the time spanning from the sampling to the analysis were deemed insufficient to cause other preanalytical errors.

Blood gas analysis was performed by inserting the syringe into the blood gas analyzer (Radiometer ABL 735 GLAXP) in a dedicated port where a fine aspiration tube withdrew the sample. Once analysis commenced, the operator entered the blood temperature into the computer. The analyzer then completed the analysis, and the results were printed.

Routine maintenance of the blood gas analyzer was scheduled and performed as suggested by the user manual, and a two-point automatic calibration system was used every 2 h.

### Statistical analysis

Statistical analysis was performed on the 32 female tortoises. This choice was made because of a numerical imbalance between the female and male tortoises sampled; a sex-related variation in blood analysis in tortoises is known and documented (9, 24), and excluding the males was deemed a better choice to have a more representative value of the analysis.

The data collected were tested for distribution using the Shapiro–Wilk W test to investigate standardized skewness and kurtosis.

A value of *ά* = 0.05 was used as the significance level. Values of *p* > 0.05 were considered indicative of a normal distribution, consistent with values of skewness and kurtosis between −2 and +2 (27, 28).

ASVCP reference interval guidelines were used to establish exploratory ranges, even though the sample size was not large enough. This choice was made so that a single guideline would be used throughout the study, although the ranges presented should be considered descriptive only.

Exploratory ranges for normally distributed data were calculated by adding and subtracting two times the standard deviation from the mean value. For non-normally distributed data, the nonparametric method was used, and exploratory reference intervals were established as the 5th and 95th percentiles (29). Frequency histograms were constructed to graphically display the data collected.

Even though only female tortoises were included in the exploratory range determination, analysis and comparison of the results with those obtained from the male group were still performed. This choice was made to perform an exploratory evaluation of the potential concordance of the results between the two sexes, despite the great numerical differences between the two sample groups. The Mann–Whitney *U* test was used (30), which was deemed appropriate because of the sample size and the distribution of the data. Confidence intervals were subsequently obtained, and the results were graphically displayed using box plots.

The author performed visual comparisons between obtained values and literature values without statistical analysis. This approach was chosen due to (1) high variability among species reported in the literature, (2) use of different analyzer types across studies, (3) lack of analyzer validation for the tested species, and (4) the exploratory nature of this study.

## Results

### Blood gas analysis

Blood gas analysis was performed on all 38 animals.

**Figure 1:**
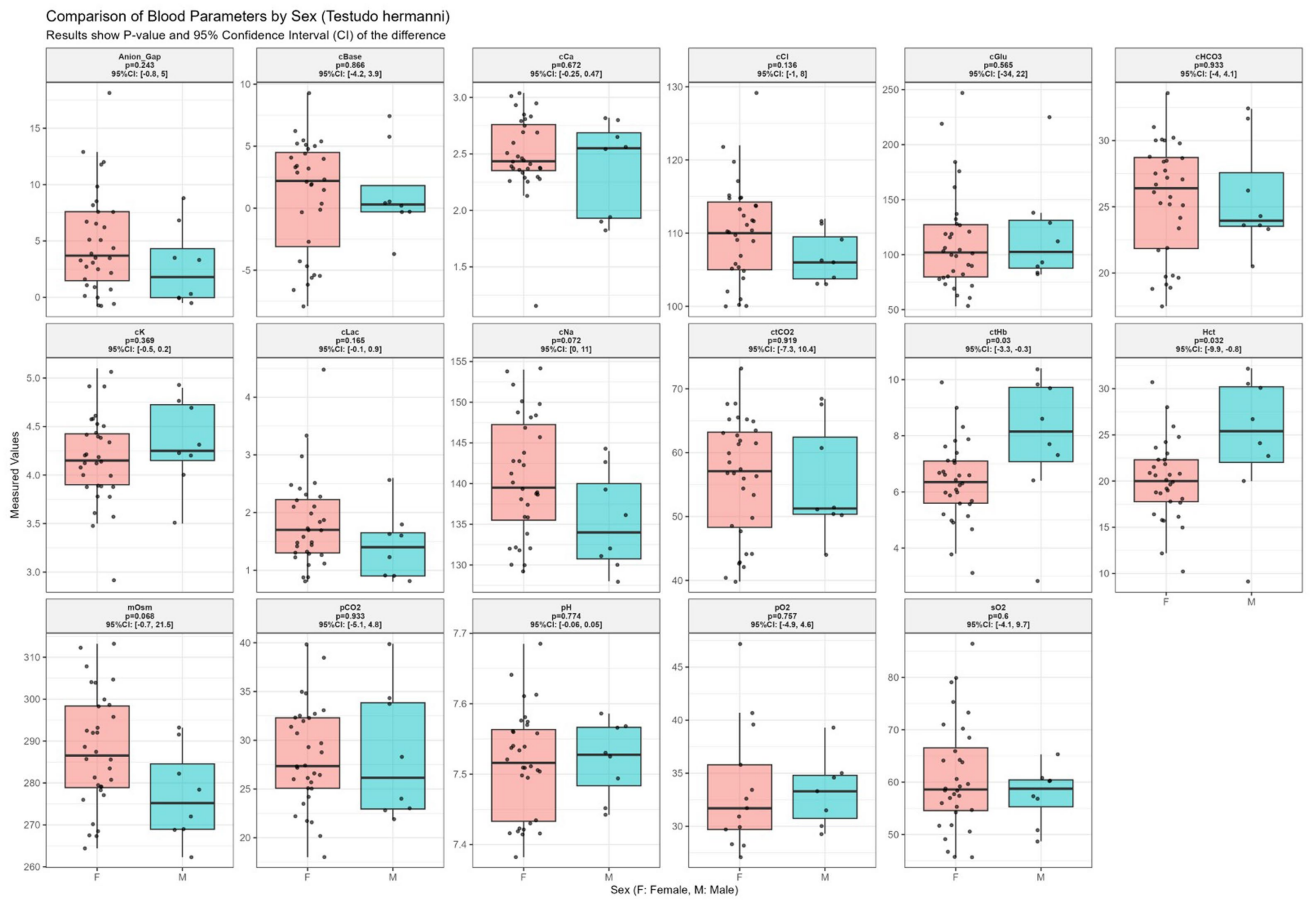
Comparison of blood parameters by sex in Testudo hermanni. Each panel displays the boxplots and individual data points for the evaluated analytes. For each parameter, the *p*-value and the 95% Confidence Interval (CI) of the difference are reported in the header. Data for female (F) and male (M) tortoises are respectively represented by pink and light blue box plots.

Owing to the disparity between the number of females and males, two separate analysis groups were formed: one with only females (*n* = 32) and the other with only males (*n* = 6). The data reported refer to the group consisting of females (*n* = 32) since the number of males was too low. Data obtained from the male (*n* = 6) group were compared with those from the female group to investigate potential agreement between the two groups. The results of this comparison are graphically displayed using box plots in Figure 1.

Seventeen analytes were evaluated (pH, pCO₂, pO₂, ctHb, sO₂, Hct, cK^+^, cNa^+^, cCa^++^, cCl^−^, cGlu, cLac, cBase, mOsm, cHCO₃^−^, ctCO₂, and anion gap); twelve of those analytes were normally distributed (pH, pCO₂, pO₂, ctHb, sO₂, Hct, cK^+^, cNa^+^, cCl^−^, mOsm, cHCO₃^−^, and ctCO₂), and five (cCa^++^, cGlu, cLac, cBase, and anion gap) were not normally distributed.

Data for all analytes, consisting of the units of measurement (UM), mean, median, standard deviation (SD), exploratory range (ER), lower exploratory range with 90% confidence intervals (LER CI 90%), upper exploratory range with 90% confidence intervals (UER CI 90%), and distribution (D), listed as Gaussian (G) or non-Gaussian (nG), are presented in Table 1.

**Table 1:** Venous blood gas analytes, both measured and calculated, of female *Testudo hermanni*, consisting of the unit of measurement (UM), mean, median, standard deviation (SD), exploratory range (ER), lower exploratory range with 90% confidence intervals (LER CI 90%), upper exploratory range with 90% confidence intervals (UER CI 90%), and distribution (D), listed as Gaussian (G) or non-Gaussian (nG).

Analyte	UM	Mean	Median	SD	ER	LER (CI 90%)	UER (CI 90%)	D
pH	–	7.52	7.52	0.08	7.36–7.68	7.34–7.38	7.66–7.7	G
pCO₂	mmHg	28	27	5.2	17.6–38.4	16.1–19.1	36.9–39.9	G
pO₂	mmHg	34	32	6	22–46	20–24	44–48	G
ctHb	g/dL	6.4	6.4	1.4	3.6–9.2	3.2–4	8.8–9.6	G
sO₂	%	60	59	10	40–80	37–43	77–83	G
Hct	%	20.0	20.0	4.2	11.6–28.4	10.9–12.3	27.7–29.1	G
cK^+^	meq/L	4.2	4.2	0.5	3.2–5.2	3.1–3.3	5.1–5.3	G
cNa^+^	meq/L	141	140	7	127–155	126–128	154–156	G
cCl^−^	meq/L	110	110	7	96–124	96–98	122–126	G
mOsm	mmol/kg	290	290	14	262–318	258–266	314–322	G
cHCO₃^−^	mmol/L	26.0	26.0	4.3	17.4–34.6	16.2–18.7	33.4–35.9	G
ctCO₂	Vol%	56	57	9	38–74	35–41	71–77	G
cCa^++^	meq/L	2.5	2.4	0.4	2.2–3.0	2.1–2.3	2.9–3.1	nG
cGlu	mg/dL	112	102	48	62–200	48–76	186–214	nG
cLac	mmol/L	1.8	1.7	0.9	0.9–3.1	0.6–1.2	2.8–3.4	nG
cBase	mmol/L	1.0	2.2	7.6	−6.4 to 5.8	−8.6 to −4.2	3.6–8.0	nG
Anion gap	meq/L	4.9	3.7	6.1	−0.6 to 12.4	−2.4–1.2	10.6–14.2	nG

## Discussion

Blood gas parameters were evaluated to establish reference ranges and characterize the clinical pathology of *Testudo hermanni*. The number of tortoises examined was insufficient to calculate reference ranges for the ASVCP guidelines for the studied analytes (29); thus, the same statistical methods were applied, but the values obtained can only be considered explorative.

The values obtained were compared with data found in the literature for *Testudo hermanni* when present; comparisons were also made with other chelonian species, both when data were present in recent literature and when data regarding *Testudo hermanni* were not present. Owing to the exploratory nature of the data presented in this study, comparisons were not supported by statistical analysis and were kept at a descriptive level, as the results presented are meant to provide guidelines for future investigations rather than to provide outright clinically relevant information.

Five of the seventeen analytes (pO₂, sO₂, Hct, ctHb, and ctBil) were considered unreliable by the authors. Values were nonetheless obtained and compared with others found in the literature, but their relevance and interpretations must take that unreliability into account.

PO₂ in chelonian blood showed a nonlinear regression, which made it incompatible with the automatic correction based on temperature performed by the analyzer (31). Other authors have proposed and used manual correction for this analyte and for pH, pCO₂, and cHCO₃^−^ (8, 12, 32, 46, 48). pH and pCO₂, in contrast with pO₂, were linearly related; this made them suitable for the automatic correction performed by the analyzer, as the same type of regression is performed for human blood (31). Other authors investigating chelonian venous blood gases have obtained pH and pCO₂ values that, after the automatic temperature correction was performed by the analyzer, were not significantly different from those obtained through manual correction (12, 32). The same results were not obtained for pO₂, which significantly differed. Thus, the authors considered the pO₂ values to be unreliable and decided to use the pH and pCO₂ values without further manual correction in addition to that performed by the analyzer, considering that the equations used by the analyzer were almost the same as those used in the literature for manual correction (12, 32).

sO₂ should be considered unreliable because testing was performed on venous samples, and the study of venous sO₂ compared with that of arterial sO₂ is not considered useful (33).

The Hct value determined by a blood gas analyzer is usually calculated and not measured and, to determine that value, ctHb or the conductivity of a blood sample is usually utilized. First, the analyzer determines Hct using an equation based on the linear relationship between Hct and ctHb (34). Then, blood sample conductivity is measured, as red blood cells are insulating; thus, conductivity is inversely correlated with Hct (34). The analyzer used in this study uses the conductivity method to calculate Hct. The tendency of blood gas analyzers to underestimate Hct has been previously reported (35), and this may be due to the larger volume occupied by the nucleated red blood cells, which would change the relationship with blood sample conductivity related to the hematocrit; thus, a different calculation is needed for correct determination rather than using that derived from human or mammalian red blood cells.

With respect to ctHb, as different types of Hb are present in reptilian blood, which differs from mammalian blood and was never tested with a POC blood analyzer (36, 37), measurements obtained for this value are not necessarily unreliable but should be considered with extreme caution. Spectrophotometry was used in the blood gas analyzer to assess ctHb, and differences in reptile hemoglobin light refraction that have not yet been sufficiently explored could influence the determination of this parameter.

Last, ctBil was not considered reliable because of the lower detection limit of the analyzer used, which only evaluates ctBil if this parameter is already in the pathological range.

To the author’s knowledge, no standardized protocol have been suggested for blood gas analysis in chelonian or reptilian species. As such, in the realization of this study, good practice and sample management used in mammalian medicine were involved, but a better understanding of reptile variability in the execution of this type of test, such as preferred sampling site or lymph interference, should be explored, and this study serve as a starting point to develop that knowledge.

Eight analytes, namely pH, pCO₂, pO₂, cK^+^, cNa^+^, cCl^−^, cGlu, and mOsm, were compared to values for *Testudo hermanni* obtained from the literature (13, 23, 38, 39). The pH was found to be similar; pO₂ and mOsm were similar and had a narrower range than those found for comparison; cK^+^, cNa^+^, and cCl^−^ were similar; while pCO₂ and cGlu were found to be different from their corresponding values.

Notably, the values reported in the literature are mostly not obtained through blood gas analysis but rather through biochemical analysis (23, 38, 39); thus, a spectrophotometry method is used rather than electrodes and membranes, as used by a blood gas analyzer. This could be relevant for analytes that did and did not show accordance. No prior study has compared blood gas analyzer and biochemistry analyzer results for *Testudo hermanni*. Although our visual comparison lacks statistical validation, it provides a preliminary basis for future systematic comparisons and validation studies. With respect to analytes that differed, the different methods used by the analyzers could play a relevant role; this could be especially true for cGlu, for which values reported in the literature were more similar to each other than to the values obtained in this study.

Thirteen analytes, namely pH, pO₂, pCO₂, sO₂, cK^+^, cNa^+^, cCa^++^, cCl^−^, cGlu, cBase, cHCO₃^−^, ctCO₂, and cLac, were compared to values reported for the green turtle (*Chelonia mydas*) (11, 12), Negev tortoise (*Testudo werneri*) (7), Kemp Ridley’s turtle (*Lepidochelys kempii*) (12), red-footed tortoise (*Chelonoidis carbonarius*) (10), and common box turtle (*Terrapene carolina*) (6).

The pH, cBase, and cCl^−^ values were similar to those of the other samples, showing minimal differences.

The PO₂ values were quite similar to those obtained for *Chelonia mydas* and *Lepidochelys kempii* (12) but not very similar to values obtained from other studies. Considering the lack of manual temperature-based correction for that analyte in this study, the discrepancies with data found in the literature were to be expected, as the studies cited all used a manual correction for that analyte. The similarities found are more likely to be attributed to coincidence than to effective concordance, and the results presented in this study regarding pO₂ should be considered with regard to the lack of a warranted manual temperature correction.

The PCO₂ values were similar and had a narrower range than those presented for *Chelonoidis carbonarius* and *Terrapene carolina* (6, 10) but different from those obtained for other chelonians. The fact that values obtained from tortoises and not turtles were closest could be due to the different physiological adaptations to diving and submersion; although terrestrial chelonians are more reliant on anaerobic metabolism than mammals are, marine chelonians, owing to their natural environment, could have developed blood buffers with different efficacies and could be more reliant upon non-respiratory buffers, as those are not dependent upon emersion. Further data and statistical confirmation are warranted. With respect to the temperature correction of pCO₂, the studies that presented similar values both used a manual correction for the analyte; the similarities between those values and those obtained in this study, which used no manual correction, are considered to be in accordance with the fact that automatic temperature-based correction performed by the analyzer is adequate for pCO₂.

Another analyte that was similar to the values reported in the literature was cK^+^; the unit of measurement, which is often presented in other studies, differs from that used in this study, but the two different units should be, in the case of this analyte, considered interchangeable (40).

Among the other analytes, cNa^+^, cHCO₃^−^, and cLac were found to be similar to only one of the ranges to which they were compared: cNa^+^ was similar to one of the values reported for *Chelonia mydas* (11), and cHCO₃^−^ was similar to the values reported in another study for *Chelonia mydas* (12), whereas cLac was similar to and had a narrower range than that reported for *Terrapene carolina* (6). Differences in cLac values likely reflect the distinct environmental pressures on each chelonian group. Marine species diving in hypoxic conditions may require enhanced respiratory compensation mechanisms that alter blood buffer management and increase lactate tolerance compared to terrestrial species. For cHCO₃^−^, the discrepancies could be due to a lack of manual temperature-based correction, as there is no evidence of the type of regression associated with that analyte; thus, the automatic temperature correction performed by the analyzer could have been unsuitable.

CtCO₂ was reported in a unit of measurement that was not compatible with those found and listed in the literature.

The other analytes differed from the ranges found. SO₂, as previously stated, is considered to be of minimal relevance because of the type of blood sample used; values are displayed for explorative purposes, but their real usefulness must be more clearly researched. CGlu has a lower end of the range similar to that found in *Chelonoidis carbonarius* (10), although the upper end is higher than any other range found. The minimal similarity of the lower end to another tortoise, rather than a turtle, could also lie in the difference in marine lifestyle, although true biological differences seem to be the more likely cause of the variability found.

cCa^++^ was found to differ from all other results reported in the literature: the closest value was the upper limit found for *Chelonoidis carbonarius* (10). Although upper and lower limits varied widely across studies, some ranges showed similar spans (7). These differences likely reflect true biological variation among populations. Additionally, client-owned tortoises experience more controlled conditions, such as standardized feeding, than free-living individuals, which may have resulted in narrower parameter ranges.

A single analyte, anion gap, was impossible to compare because of the lack of sources with which it can be compared.

Comparisons of the values obtained for females with those obtained for the six males revealed that only ctHb and Hct were significantly different. The great disparity between the two sample sizes still hinders any type of conclusion regarding sex-related differences, but the data displayed still provide a useful starting point for future evaluations.

### Limitations and future directions

This study has several limitations, which are largely related to its methodology. Those constraints represent not only important considerations needed for a critical evaluation of data presented but also highlight future steps needed to expand chelonian blood gas knowledge.

The first limitation is that the analyzer utilized for this study is not validated for the species or for any other reptile species; therefore, the data obtained in this study must be considered strictly exploratory. To the best of the authors’ knowledge, no blood gas analyzer has been validated for reptilian species. Validation studies can be found for ectothermic species, but they involve primarily fish (35). Nevertheless, blood gas was and is still being investigated in reptiles, especially in chelonians. Validation of a blood gas analyzer was beyond the scope of this study, but it is still a goal which, in this author’s opinion, must be addressed, and any additional data provided could help reach the point at which a validation study can be performed.

Another limitation of this study is the sampling site: although samples with macroscopically visible lymph dilution were discarded, the sampling site used (dorsal subcarapacial cervical plexus) is reported to have an increased likelihood of lymph dilution even if not macroscopically visible (41). Not all analytes examined in this study have a known effect on lymph dilution, and investigations on the effects of lymph in blood samples have been performed with blood biochemistry and not blood gas analysis (42, 43). Values of cCl^−^ and cGlu were described as not influenced by lymph contamination, and cNa^+^ was described as not influenced in one study (42) and influenced, but not in a clinically relevant way, by another study (43). cK^+^ was described as influenced in a clinically relevant way only when the percentage of lymph dilution was equal to or greater than 25% (43). Although some analytes have been reported as unaffected by lymph dilution, this finding may not apply uniformly across measurement methods. Blood gas analyzers using electron or membrane-based detection may respond differently to lymph dilution than spectrophotometric methods do. Thus, the effects of lymph dilution on blood gas parameters must be more thoroughly investigated, for example, by using jugular vein blood samples to obtain new values and comparing values obtained from two different sampling sites. Furthermore, a better understanding of lymph dilution in the determination of analytes through electron and membrane method could prove useful as, if it would produce results less influenced than spectrophotometry, it could lead the way to the determination of new and more reliable methods of obtaining blood parameters.

Another possible limitation is the use of the temperature obtained from the prefemoral fossa instead of that obtained using a rectal probe. Although it has been reported to be a valid approximation of body temperature (12, 39), values should be studied with temperature correction based on rectal temperature, and a comparison between the two methods of temperature measurement should be made.

Another limitation regarding the methodology is the use of preheparinized syringes and the type of heparin used: the syringes used for this study were not preheparinized but were heparinized manually by an operator. This could lead to imbalances in the electrolyte analysis. Further investigations with benchtop analyzers using balanced preheparinized syringes are warranted.

Studies involving chelonian blood gases primarily use an i-STAT blood gas analyzer and not a benchtop analyzer because of the importance given to the on-field research aspect; the i-STAT was validated for an ectotherm species (31), but some biased results have been reported between i-STAT and other non-POC analyzers (44, 45). Although this has to be considered a limitation, especially with respect to comparisons with values obtained in other studies, the fact that benchtop analyzers are considered the gold standard in blood gas analysis must be acknowledged, and further studies using this type of analyzer are paramount to further develop our knowledge in the field.

The use of only female tortoises in this study is a further limitation, as is the lack of seasonal difference across the sample times. The unequal sex ratio prevented analysis of sex-dependent variation in the parameters investigated. This limitation is noteworthy because sex is known to influence blood biochemistry values in chelonians (9, 23). This limitation led to the inability to consider values obtained as representative for *Testudo hermanni* in general but could prove useful if paired and confronted in the future with new measurements from samples of both sexes and from sex-specific samples to help in further exploration of sex-related differences and to serve as a foundation for future blood gas parameters investigation in this species.

With respect to sex-associated differences, seasonally associated variations in the clinical pathology of chelonians are also known and documented. The sampling time and temperatures at which the subjects were exposed were too few and too similar to try to establish season- or temperature-related variations. Further investigations involving the performance and comparison of samples at different times of the year are warranted.

## Conclusion

This study investigated and provided descriptive blood gas analysis data for *Testudo hermanni* and calculated exploratory ranges for seventeen parameters (pH, pCO₂, pO₂, cTHB, sO₂, Hct, cK^+^, cNa^+^, cCa^++^, cCl^−^, cGlu, cLac, cBase, mOsm, cHCO₃^−^, ctCO₂, and anion gap). This study should be useful to enhance and expand knowledge of blood gas analysis and clinical pathology of chelonians.

## Data Availability

The raw data supporting the conclusions of this article will be made available by the authors without undue reservation.
